# Activation of coagulation and tissue fibrin deposition in experimental influenza in ferrets

**DOI:** 10.1186/1471-2180-14-134

**Published:** 2014-05-30

**Authors:** Marco Goeijenbier, Eric CM van Gorp, Judith MA Van den Brand, Koert Stittelaar, Kamran Bakhtiari, Joris JTH Roelofs, Geert van Amerongen, Thijs Kuiken, Byron EE Martina, Joost CM Meijers, Albert DME Osterhaus

**Affiliations:** 1 Department of Viroscience laboratory, Erasmus MC, room ee1671, Rotterdam, CE 50 3015, The Netherlands; 2 Viroclinics Biosciences BV, Rotterdam, The Netherlands; 3 Department Experimental Vascular Medicine, Academic Medical Center, Amsterdam, The Netherlands; 4 Department Pathology, Academic Medical Center, Amsterdam, The Netherlands

**Keywords:** Influenza A virus (Human), H3N2 subtype, H1N1 subtype, H5N1 subtype, Consumption coagulopathy

## Abstract

**Background:**

Epidemiological studies relate influenza infection with vascular diseases like myocardial infarction. The hypothesis that influenza infection has procoagulant effects on humans has been investigated by experimental animal models. However, these studies often made use of animal models only susceptible to adapted influenza viruses (mouse adapted influenza strains) or remained inconclusive. Therefore, we decided to study the influence of infection with human influenza virus isolates on coagulation in the well-established ferret influenza model.

**Results:**

After infection with either a seasonal-, pandemic- or highly pathogenic avian influenza (HPAI-H5N1) virus strain infected animals showed alterations in hemostasis compared to the control animals. Specifically on day 4 post infection, a four second rise in both PT and aPTT was observed. D-dimer concentrations increased in all 3 influenza groups with the highest concentrations in the pandemic influenza group. Von Willebrand factor activity levels increased early in infection suggesting endothelial cell activation. Mean thrombin-antithrombin complex levels increased in both pandemic and HPAI-H5N1 virus infected ferrets. At tissue level, fibrin staining showed intracapillary fibrin deposition especially in HPAI-H5N1 virus infected ferrets.

**Conclusion:**

This study showed hemostatic alterations both at the circulatory and at the tissue level upon infection with different influenza viruses in an animal model closely mimicking human influenza virus infection. Alterations largely correlated with the severity of the respective influenza virus infections.

## Background

There are three manifestations of influenza in humans: seasonal, avian and pandemic influenza. Seasonal influenza is caused by influenza A or B viruses which infect 5-15% of the human population every year [1,2]. Symptoms vary from mild respiratory complaints to fatal respiratory distress due to multiple organ failur. Symptoms depend largely, however, on the health and immune status of the infected individual and the pathogenicity of the specific virus involved. While avian influenza A viruses cause sporadic zoonotic infections in humans, that do not spread efficiently among humans [1], these infections may result in respiratory disease manifestations that range from mild to fatal, which among other variables largely depends on the virulence of the virus involved. Although most seasonal influenza virus infections are self-limiting, they do cause a considerable burden of disease that may be aggravated by complications of the infection [3]. Patients with chronic illness are particularly at risk of developing these complications when suffering from (seasonal) influenza, like the observed increased risk for developing cardiovascular disease during or shortly after influenza virus infection [4]. This observation is supported by the results of two intervention studies which showed a risk reduction of myocardial infarction after influenza vaccination, which later was confirmed by a meta-analysis carried out among 292,383 patients. This analysis showed significant reductions in myocardial infarction, all-cause mortality, and major adverse cardiac events in the influenza vaccinated groups [5-7]. However, the etiological pathway and the frequency by which influenza predisposes for clinically relevant thrombotic disease has yet to be determined. Current data suggest that influenza virus infection causes an unbalanced coagulation manifested by a procoagulant state (for review see [8-11]). Indications for this increased clotting tendency have come from clinical, experimental mouse and *in vitro* data. Clinical reports range from mild increased coagulation and fibrinolysis markers such as von Willebrand factor (VWF) and D-dimer levels, to disseminated intravascular coagulation observed in severe avian influenza [12-14]. Experimental mouse data indicate a procoagulant state characterized by increased thrombin generation, fibrin deposition, and an impaired fibrinolysis [15,16]. However, as the mouse is not a natural host to influenza virus, mouse influenza models use mouse-adapted influenza viruses which cause a disease quite different from that of human influenza [17]. Collectively experimental animal studies and epidemiological data have largely remained inconclusive and cannot readily be translated into clinically relevant conclusions.

The laboratory ferret (*Mustela putorius furo*) is not only susceptible to human isolates of seasonal, avian and pandemic influenza viruses, but pathogenesis and severity of the respective clinical manifestations of these infections are to a large extent similar to those found in humans [18,19]. Therefore, to address the hypothesis that humans at risk for vascular disease may develop clinically overt vascular thrombosis during or shortly after influenza virus infection [20], we collected plasma samples during a time course pathogenesis experiment in which ferrets were infected with seasonal-, avian- or pandemic influenza viruses [21]. Even though ferrets are not generally considered to represent the high risk patients for vascular thrombotic disease, they do offer a biologically variable and reliable animal model to address the activation of coagulation during influenza virus infection. Prothrombin time, activated partial thromboplastin time, von Willebrand factor (VWF) activity, D-dimer levels, and thrombin-antithrombin complexes were measured in sequentially collected plasma samples. In addition fibrin staining was carried out on the lungs of infected animals upon euthanasia to address the coagulation status at the tissue level. All these parameters were evaluated in relation to virological parameters and data on disease severity.

## Results

### Clinical signs, pathology and virology of ferrets after infection with H3N2-, pH1N1- or highly pathogenic H5N1 avian – influenza viruses

Clinical signs, pathological changes and virological parameters of this time course experiment in ferrets have been reported previously [21]. Data important for this study are summarized in Table 1. In short, clinical signs varied greatly between the three influenza virus and mock infected groups. All animals infected with H3N2, pH1N1, or mock infection, survived the infections. H3N2 virus infected ferrets showed mild clinical signs; nasal discharge, sneezing, decreased tendency to eat, and bodyweight decrease by 11% (SD 8.5-13%) at 7 dpi. Detection of infectious virus was restricted to the nose and peaked at 1 dpi. Upon necropsy the lungs of the H3N2 infected ferrets showed up to 10% consolidation by gross pathology while the relative lung weights did not differ from the controls.

**Table 1:** **Overview of the clinical data (bodyweight decrease, relative lung weight, lung damage) and virological parameters (virus titers) partly adapted from Van den Brand et al. 2012 Plos One**[21]

**Day**		**1**	**2**	**3**	**4**	**7**	**14**
Bodyweight	H3N2	-51	-100	-69	-124	-186	-205
		(16–86)	(9–190)	(33–104)	(117–130)	(141–231)	(101–309)
	pH1N1	-68	-169	-142	-250	-251	-193
		(22–114)	(161–176)	(74–210)	(185–315)	(190–312)	(19–368)
	H5N1	-70	-131	-170	-190	┼	┼
		(35–105)	(112–149)	(142–198)	(135–246)		
	Control	-44	-20	+7	-34	-62	-46
		(31–57)	(+30 - -69)	(+40- -25)	(+19 - -88)	(+10 - -134)	(+30 – 123)
Relative lung weight 10^-2^ gram	H3N2	0.6	0.6	0.6	0.6	0.6	0.6
		(0.5-0.7)	(0.6-0.7)	(0.5-0.6)	(0.5-0.7)	(0.6-0.7)	(0.5-0.6)
	pH1N1	0.8	1.0	1.1	1.2	1.3	0.7
		(0.7-0.8)	(0.9-1.2)	(0.9-1.2)	(1.1-1.3)	(1.0-1.6)	(0.6-0.8)
	H5N1	0.9	1.4	1.7	2.4	┼	┼
		(0.6-1.2)	(1.1-1.7)	(1.2-2.2)	(2.0-2.7)		
	Control	0.7	0.7	0.6	0.6	0.6	0.6
		(0.7-0.8)	(0.6-0.8)	(0.6-0.6)	(0.6-0.7)	(0.6-0.7)	(0.5-0.7)
Lung damage %	H3N2	3.8	2.5	0	0	0	1.3
		(0–8.5)	(0–5.4)				(0–3.8)
	pH1N1	22.5	25.0	40.0	45.0	47.5	25.0
		(17.5-27.5)	(19.2-30.8)	(31.8-48.1)	(35.0-55.0)	(30.4-64.6)	(19.2-30.8)
	H5N1	25.0	55.0	62.5	77.5	┼	┼
		(12.1-37.9)	(35.9-74.2)	(40.3-84.7)	(55.3-99.7)		
	Control	3.8	6.3	6.3	1.3	5.0	3.8
		(1.3-6.3)	(1.5-11)	(1.5-11)	(0–3.8)	(5–5)	(1.3-6.3)
Turbinates/nasal concha log TCID_50_	H3N2	7.0	6.3	5.1	4.8	neg	neg
		(5.5-8.5)	(5.4-7.3)	(3.9-6.2)	(3.4-6.1)		
	pH1N1	8.2	8.0	7.6	7.0	neg	neg
		(8.0-8.5)	(7.7-8.3)	(7.0-8.2)	(6.2-7.9)		
	H5N1	4.8	5.0	5.6	4.9	┼	┼
		(3.5-6.1)	(4.4-5.6)	(4.1-7.0)	(3.4-6.4)		
Trachea log TCID_50_	H3N2	2.4	neg	neg	neg	neg	neg
		(<1.7-3.1)					
	pH1N1	5.5	5.4	5.9	5.5	neg	neg
		(5.0-6.0)	(5.0-5.9)	(5.6-6.3)	(4.3-6.9)		
	H5N1	5.5	4.7	5.1	4.7	┼	┼
		(4.7-6.3)	(4.2-5.1)	(4.1-6.2)	(3.4-6.0)		
Lung log TCID_50_	H3N2	neg	Neg	Neg	neg	neg	neg
	pH1N1	7.5	5.2	5.5	5.6	neg	Neg
		(7.2-7.8)	(4.7-5.8)	(5.1-6.0)	(5.1-6.2)		
	H5N1	6.6 (6.0-7.2)	5.2 (4.7-5.6)	5.8 (5.5-6.1)	5.2 (4.7-5.6)	┼	┼

Bodyweight decrease (+/- SD), relative lung weight, lung damage and viral titers (log TCID_50_ +/- SD) for lung, turbinates and trachea over time in H3N2, pH1N1 and H5N1 infected ferrets and the control (mock infection). Weight in grams, Lung damage in relative percentage of total lung tissue. Relative lung weight was calculated by lung weight divided by bodyweight times 100%. Infectious virus titers in log_10_ TCID_50_/g threshold were based on Mock control and <1.8 for turbinates, <1.9 for trachea, <1.4 log_10_ TCID_50_ for lung.

Ferrets infected with the pH1N1 virus showed more severe clinical signs compared to the seasonal H3N2 virus infected ferrets, with a body weight decrease around 15% (SD 11.4-18.6%). Viral titers during pH1N1 virus infection also peaked at 1 dpi, but occurred at similar levels throughout the whole respiratory tract. One ferret in the pH1N1 group developed severe dyspnea. Relative lung weights increased compared to those of the mock infected animals starting from day 1. Their relative lung weights (weight of lung divided by bodyweight multiplied by 100) had increased from 0.6% (SD 0.57-0.65) to 1.3% (SD 1.0-1.6). The lungs of the pH1N1 virus infected ferrets showed up to 70% consolidation by gross pathology.

The HPAI-H5N1 virus infected ferrets showed more severe clinical signs with dyspnea leading to hypoxia. On 2.5 dpi, one animal died and one animal was euthanized for ethical reasons. On 3 dpi, another animal died before it could be euthanized. H5N1 virus was predominantly found in the alveoli and viral titers peaked for a longer period, from 1 to 3 dpi. Upon necropsy the lungs of the H5N1 infected ferrets showed up to 100% consolidation by gross pathology and relative lung weight was increased up to 2.78% of total body weight, while pre-inoculation samples had a mean relative lung weight of 0.66%. Mock infected ferrets showed no significant clinical signs or weight loss. Only minor consolidations in about 10% of the lung tissue were found upon necropsy.

To assess a potential link between hemostatic alterations with total virus titers we generated the areas under the curve (AUC) from the virus titer as shown in Table 2.

**Table 2:** Viral parameters for correlation tests with coagulation results from 0.5-4 dpi

**Virus**	**Day**	Virus titer*	**Lung virus AUC#**	**Respiratory tract AUC#**
**H3N2**	0.5	3.5 (2.9-4.2)	neg	0
	1	7.0 (5.5-8.5)	neg	2.6
	2	6.3 (5.4-7.3)	neg	9.3
	3	5.1 (3.9-6.2)	neg	15
	4	4.8 (3.4-6.1)	neg	19.9
**pH1N1**	0.5	26.0 (24.3-27.7)	0	0
	1	31.7 (31.1-32.3)	3.6	14.4
	2	27.0 (26.4-27.6)	10.0	43.8
	3	27.0 (25.7-28.4)	15.4	70.8
	4	25.7 (23.4-28.0)	20.1	97.1
**H5N1**	0.5	22.3 (19.5-25.2)	0	0
	1	27.61 (24.4-30.8)	3.1	12.5
	2	24.8 (22.3-27.3)	9.0	38.7
	3	26.1 (22.0-30.8)	14.5	64.3
	4	26.0 (23.9-28.0)	19.9	90.5

*Total virus titer in log TCID_50_ (cumulative titers of all organs with significant virus titers: “lung, nasal concha, trachea, bronchus and bronchial lymph nodes”) (+/- SD).

# AUC was calculated from virus titers curves. 7 dpi and 14 dpi were excluded from the analysis because we data points from 5 & 6 dpi are not available potentially resulting in over or underestimation of the true AUC.

### Both prothrombin time and activated partial thromboplastin time show transient prolongations during influenza virus infection in ferrets

**Figure 1:**
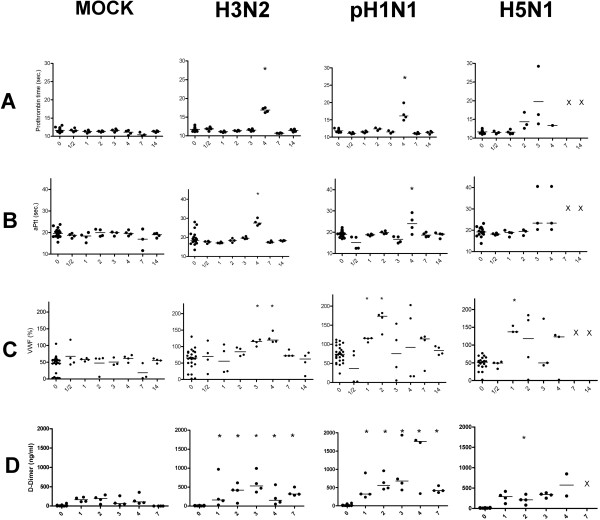
**PT (row A), APTT (row B), VWF activity (row C) and D-dimer levels (row D) in ferrets infected with mock, H3N2-, pH1N1- or H5N1 influenza virus.** Asterisk represents a p value < 0.05 in the paired samples (t = 0) or compared to the mock infection at the same time point. All influenza variants lead to (transient) increases in PT and APTT. Differences were especially observed on day 4 post infection For PT 18 and for APTT 22 out of 208 samples could not be tested due to due to technical failure or insufficient plasma volumes. VWF increase is seen in all three influenza virus groups, especially early after infection in pH1N1 and H5N1 virus infected ferrets with statistically significant results in the earliest time points after infection. D-Dimer levels were raised in all 3 influenza groups with the highest levels seen in the pH1N1 virus infected ferrets. X represents no data available since for H5N1 on day 7 and 14 no ferrets were alive.

To evaluate tissue factor pathway activation of the coagulation cascade we tested the prothrombin time (PT) for all samples. Before inoculation all ferrets had PTs within normal range.Figure 1 (row A) summarizes the PT results over time for all four groups. For both the H3N2 virus and pH1N1 virus groups, PT values increased with approximately 4 seconds at 4 dpi compared to pre-inoculation samples (H3N2 p = 0.001, pH1N1 p = 0.02) and the mock infected animals at the same day (H3N2 p = 0.03, pH1N1 p = 0.03). In the H5N1 infected ferrets, PT prolongation started at 2 dpi with a prolongation up to 16 seconds in individual animals. A clear trend is seen with PT increasing up to 30 seconds at 3 dpi. On multiple occasions ferrets died before samples could be drawn, consequently the data depend on a small number of observations with a potentially strong survival bias. On 4 dpi only one sample met the quality criteria for PT testing in the H5N1 group with a PT of 13.4 seconds, a 1.4 second increase compared to mean + SD from day 0 and mock samples (+/- SD). No significant changes in PT were observed over time in the mock infected group. Row B in Figure 1 shows the Activated partial thromboplastin time (APTT) a measurement of the intrinsic pathway of coagulation. APTT´s showed similar trends as PT´s. At 4 dpi, APTT´s were prolonged in all the three infected groups (Figure 1). H3N2 virus and pH1N1 virus infected ferrets showed mean APTT’s of 27.8 (26.1-29.6) and 24 (19.6-28.4) seconds respectively while for the mock group this was 19.7 (18.5-20.9) seconds. Paired testing showed that the pH1N1 virus infected ferrets had significantly prolonged APTT’s than the samples from pre inoculation (p = 0.02). No significant difference was seen compared to the mock infected group, potentially due to lack of power. Comparing 4 dpi samples with all pre-inoculation samples results in significant differences for both H3N2 and pH1N1 (H3N2 p = 0.001 pH1N1 = 0.02). Three out of four ferrets inoculated with H3N2 and sacrificed at 4 dpi already showed APTT prolongation before inoculation. This was not observed in any of the other pre-inoculation samples, but hampers the interpretation of the significant lengthening on 4 dpi compared to the mock infected group (p = 0.03) resulting in a non-significant result in paired sample testing. HPAI-H5N1 virus infected ferrets showed a trend toward prolonged APTT on 3 dpi with a mean of 28 (17.1-38.9) seconds and on 4 dpi 26.3 (17.3-25.3) seconds, which was statistically significant when compared to all APTT results in pre inoculation samples (3 dpi p = 0.02, 4 dpi p = 0.02) .

### Increased Von Willebrand factor activity during influenza virus infection in ferrets suggests endothelial cell activation

To study endothelial cell activation Von Willebrand Factor activity (VWF) was measured. Figure 1 (row C) summarizes the results indicating that, compared to mock infection, VWF activity tends to early increase in all three influenza virus infected groups. H3N2 virus infected ferrets showed increased VWF activity from 2 dpi onward. Significant differences were observed at 2, 3 and 4 dpi compared with mock infected ferrets on the same time points (2, 3 & 4 dpi, p = 0.028). Compared to all day 0 samples, drawn before inoculation, Mann Whitney U testing shows significant results for 3 and 4 dpi (3 dpi, p = 0.004 and 4 dpi, p = 0.003). For the pH1N1 virus infected group mean VWF activity differed significantly at 1 and 2 dpi compared to all pre-inoculation samples (1 dpi, p = 0.0025, 2 dpi, p = 0.001). At these time points, VWF activity was also significantly higher compared to the pre-inoculation samples from the same ferrets in paired testing (p = 0.03). HPAI-H5N1 virus infected animals showed trends of increased VWF activity early after infection with highest levels seen at 1 (p = <0.05) and 2 dpi (p = <0.05).

### Increased D-dimer levels during influenza virus infection in ferrets confirms a procoagulant state

D-dimer levels, fibrin degradation products that are markers of both fibrinolysis and coagulation, were quantified and results are listed in row D of Figure 1. Control ferrets had relatively low D-dimer levels with a slight increase the first days after inoculation and returning to normal values at 7 dpi. This increase is most likely associated with the minor inflammation seen after inoculation with the mock cell suspension. After infection, D-dimer levels increased in all infected animals with the highest levels in the H1N1 virus infected animals (Figure 1). D-dimer levels were significantly higher in both the H3N2 and pH1N1 virus infected ferrets at all time points (H3N2 p = 0.028; pH1N1 p = 0.028) compared to the mock infected group and to the pre-inoculation samples of the same animals (H3N2 p = 0,005; pH1N1 p = 0.003). D-dimer levels remained higher, compared to mock, until 7 dpi (H3N2 p = 0.028 pH1N1 p = 0.028). HPAI-H5N1 virus infected animals showed significant increases compared to the pre-inoculation samples (p = 0.005) on 2 dpi compared to mock infected ferrets.

### Plasma thrombin-antithrombin complexes are especially increased after infection with highly pathogenic avian influenza H5N1 virus

**Figure 2:**
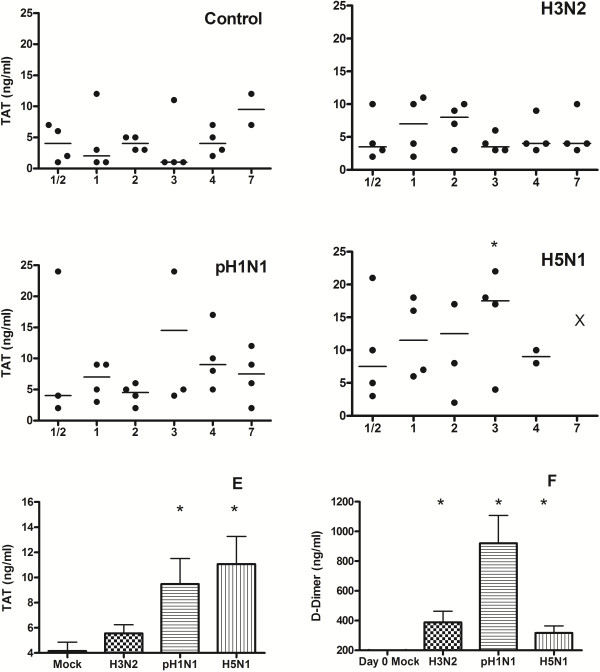
**Thrombin-antithrombin complexes in ferrets infected with mock (A), H3N2 (B)-, pH1N (C)- or H5N1(D) influenza virus.** Bar represents median in scatterdot. Asterisk represents a p value < 0.05 in the paired samples (t = 0) or compared to the mock infection. E shows mean TAT levels during the first episode of infection (day ½ to 4) F shows mean D-dimer levels during the first episode of infection (day ½ to 4). Samples drawn before infection could not be analyzed due to exogenous TAT formation during venapuncture.

To further analyze activation of coagulation all ferrets were tested for plasma thrombin-antithrombin (TAT) complexes (Figure 2). Highest TAT levels were seen in HPAI-H5N1 virus infected ferrets with a trend of increased TAT generation. To analyze the total TAT formation and compare to D-dimer formation during the course of infection we combined all data from ½ to 4 dpi of each group. This resulted in increased TAT levels for both H1N1 and HPAI-H5N1 virus infected groups (p = <0.05) and an increase in D-dimer formation during all three influenza virus infections (panel E & F Figure 2).

### Fibrin tissue staining suggests activation of coagulation at tissue level

**Figure 3:**
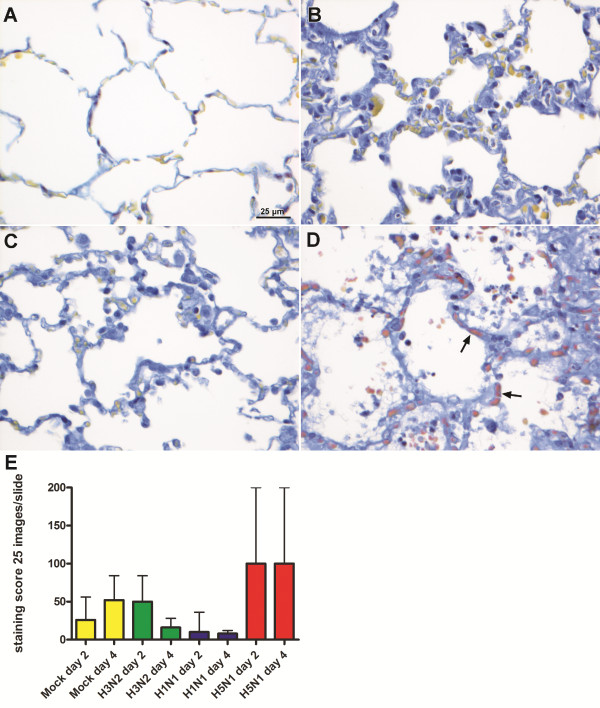
**Lendrum staining expressing fibrin (red) in lung tissue of a control ferret or 4 days after inoculation of different influenza viruses.** No staining in a non-infected ferret **(A)**, occasional intracappilairy staining of fibrin in ferrets inoculated with H3N2 **(B)** and pH1N1 **(C)**, and multifocal intracapillary staining in ferrets inoculated with H5N1 **(D)**. Panel E shows the results of a semi-quantitative scoring of fibrin deposition obtained by examining 25 images per slide.

The Lendrum staining on alveolar lung tissues showed predominantly the presence of fibrin deposition (orange-red color) in many capillaries in the alveolar walls in the HPAI-H5N1 virus infected group (Figure 3). Intermediate numbers of capillaries stained positive in the H3N2 virus infected group, a few capillaries of the pH1N1 virus infected group and in none in a negative control sample from an uninfected ferret. However, the differences did not reach statistical significance when compared to the mock infected group. The mock infected group inoculated with uninfected cell derived material did show minor signs of inflammation which were the result of intra tracheal inoculation. This resulted in an intermediate numbers of capillaries positive for fibrin staining. In the slides stained for fibrin, there is no or very little presence of fibrin in the lumen of the bronchial submucosal glands with no significant difference between the virus groups. Only in few pH1N1 and H5N1 infected animals in rare lumina of bronchial submucosal glands there was little staining of fibrin, despite the differences in inflammation within the glands between the viruses. The staining pattern in the capillaries surrounding the bronchi is similar as that in the lung parenchyma.

### Comparison of coagulation parameters with virological and disease severity data

In HPAI-H5N1- and pH1N1 virus infected animals VWF activity increased in the first two days after infection, coinciding with peak virus titers. D-dimer levels increased during the first days after infection to peak at 3 and 4 dpi, when virus titers started to significantly decrease. In these animals, highest levels in clotting times were seen at 4 dpi when a peak in relative lung weights was also observed ***.*** There was a significant correlation between multiple parameters in all three influenza groups (summarized in Table 2). Correlation analysis revealed positive correlation between PT values and AUC of the virus titers for the H3N2 virus (R = 0.8, p <0.01) and pH1N1 virus (R = 0.7, p <0.01). D-dimer levels significantly positively correlated with virus titer AUC and body weight decrease for the pH1N1 virus infected group. If we combine all data and thereby generate a dataset from influenza A virus infected ferrets, significant positive correlations can be seen between many of the virological and clinical parameters compared to the coagulation parameters. All significant R values are listed in Table 3 with those of most interest being body weight decrease with VWF, PT, APTT and D-dimer levels. Virus titer AUC’s correlated significantly with PT, APTT and D-dimer levels.

**Table 3:** Correlation between virological parameters and markers of hemostasis

**Correlation**	**H3N2**	**pH1N1**	**H5N1**	**H1N1 + H5N1**	**Influenza A**
PT -Titer total^#^	NS	-0.6 (-0.9—0.1) *	NS	-0.5 (-0.75- -0.1)*	NS
PT -AUC total^#^	0.8 (0.4-0.9)***	0.7 (0.3-0.9)**	NS	0.4 (0.1-0.7)*	0.4 (0.2-0.7)**
PT -Body weight	NS	0.8 (0.4-0.9)**	NS	0.5 (0.1-0.7)*	0.5 (0.2-0.7)**
PT -Lung weight	NS	0.6 (0.05-0.9)*	NS	NS	0.4 (0.05-0.6)*
APTT -Titer total^#^	-0.5 (-0.8 - -0.1)*	NS	NS	NS	NS
APTT -AUC total^#^	0.8 (0.6-0.9)***	NS	NS	NS	0.3 (0.05-0.6)*
APTT -Body weight	NS	0.6 (0.2-0.9)**	NS	0.5 (0.1-0.7)**	0.4 (0.2-0.6)**
APTT -Lung weight	NS	NS	NS	NS	0.3 (0.1-0.6)*
VWF-Titer total^#^	-0.6 (-0.8-0.1)*	NS	NS	NS	NS
VWF-AUC total^#^	0.7 (0.4-0.9)**	NS	NS	NS	NS
VWF-Body weight	NS	NS	NS	NS	0.4 (0.1-0.6)*
VWF-Lung weight	NS	NS	NS	NS	NS
D-dimer -Titer total^#^	NS	NS	NS	NS	NS
D-dimer -AUC total^#^	NS	0.6 (0.2-0.8)*	NS	0.5 (0.1-0.7)*	0.4 (0.2-0.6 )**
D-dimer -Body weight	NS	0.7 (0.2-0.9)**	NS	0.5 (0.2-0.7)**	0.5 (0.2-0.7)***
D-dimer -Lung weight	NS	NS	NS	NS	NS
TAT -Titer total^#^	NS	NS	NS	NS	0.3 (0.1-0.6)*
TAT -AUC total^#^	NS	NS	NS	NS	NS
TAT -Body weight	NS	NS	0.6 (0.2-0.9)*	NS	NS
TAT -Lung weight	NS	NS	NS	0.5 (0.1-0.7)**	0.3 (0.01-0.5)*

Virological parameters are listed in Table 1. For each influenza virus group coagulation values were correlated to virological and clinical parameters. This was also done for the complete influenza A group (H3N2 + pH1N1 + H5N1) and for the combination of pH1N1 and H5N1 because these two viruses are able to infect the complete respiratory tract instead of only the upper respiratory tract which is the case for H3N2.

Pearson correlation coefficients are given if the values were statistically significant. *p <0.05 **p < 0.01 ***P < 0.001 if not significant NS is listed in the table.

Using Bonferroni correction for multiple comparison significance threshold is lowered to p < 0.01. Therefore results marked with ** and *** are considered statistically significant correlations.

## Discussion

The present study demonstrates, for the first time, procoagulant effects at the circulatory and tissue level in a ferret influenza model, largely proportional to the severity of influenza virus infection. These findings are in line with earlier epidemiological, clinical, animal and *in vitro* data [6,8,13-15,20,22-24]. Ferrets have been shown to be an adequate model to study the coagulation cascade [25-27] with PT and APTT normal values varying from 11.6-12.7 and 18.9-22.3 seconds respectively. This is comparable to our 104 pre-inoculation ferret samples (PT 11.7 (+/- 0.1) and APTT 19.8 (+/- 2.2)) [26].

Like in humans, highly pathogenic avian influenza virus infection causes severe disease in ferrets, which may include bleeding complications and multi-organ failure [28,29]. In our experiments, HPAI-H5N1 virus inoculated ferrets showed severe disease, which in some cases resulted in spontaneous death. Analysis of the coagulation cascade in these animals confirmed the severity of infection with prolongation of global coagulation assays and signs of activated endothelium. PT and APTT values increased already from 2 dpi onward with individual ferrets showing an increase up to 20 seconds. This observation is suggestive for consumptive coagulopathy which is strengthened by the high levels of fibrin deposition in the lung capillaries. Consumptive coagulopathy could be the result of extreme activation of coagulation, for instance due to increased tissue factor production as is seen in other (severe) viral diseases as Ebola hemorrhagic fever [8]. The exact role for consumptive coagulopathy in highly pathogenic H5N1 infection warrants further research, but hypothetically the excess of coagulation activity could lead to microthrombosis in the pulmonary alveoli leading to respiratory distress or even multi organ failure [8]. The procoagulant changes were seen both at the tissue level and in the circulation, suggested by the TAT increase. The statistically significant increase in D-dimer levels confirms this procoagulant state. However, D-dimer levels were lower in HPAI-H5N1 virus inoculated ferrets compared to ferrets infected with H3N2 virus and especially compared to the ferrets infected with pH1N1 virus. A possible explanation for this phenomenon could be the inhibition of fibrinolysis by high levels of plasminogen-activator type 1 activity (PAI-1) during H5N1 virus infection. Unfortunately we could not test PAI-1 activity in ferret plasma with the currently available human PAI-1 activity assays. Since plasminogen is proven to play an important role in influenza pathogenesis further exploring the biology, activation and inhibition of plasminogen in influenza infection would be of great interest [30].

The second virus we used in our experiments was pH1N1. Although less severe compared to HPAI-H5N1 virus infected ferrets, pH1N1 virus infection caused severe pneumonia with lung damage in ferrets. While ferrets infected with pH1N1 virus showed remarkably high levels of D-dimer, tissue fibrin deposition was not as prominent as seen in HPAI-H5N1 virus infected ferrets. Activated coagulation in other organs than the respiratory tract or a systemic activation of coagulation could explain this phenomenon. These severe procoagulant changes in the circulation could be the result of a specific immune activation during pH1N1 virus infection. A possible explanation can be found in the work of Monsalvo et al. who showed an excessive amount of pathogenic immune complexes, which are known to have systemic procoagulant effects, in fatal pH1N1 cases [31,32]. Furthermore, TAT levels significantly increased in the first 4 days after infection and at 4 dpi there was a remarkable prolongation of PT and APTT values up to 4 seconds. The very ‘sudden’ increase of clotting times at 4 dpi is suggestive for a consumptive coagulopathy, possibly similar to what was seen in DIC due to HPAI-H5N1 virus infection and bacterial sepsis [33]. Clotting times had normalized at 7dpi, however, indicating that in contrast to bacterial sepsis, the consumptive coagulopathy is transient and less severe. The ‘sudden’ onset of clotting time prolongation may be of interest to evaluate specific coagulation factor changes during influenza infection.

To evaluate the influence of a more ‘moderate’ influenza virus infection, seasonal H3N2 virus was also included in the experiments. Although this influenza virus in general causes ‘moderate’ disease in humans and ferrets, it did cause significant procoagulant changes in the model with hemostatic alteration comparable to those of pH1N1 virus infected ferrets. However, TAT levels did not increase suggesting a more moderate procoagulant state compared to H1N1- and H5N1 virus infected animals.

Since the ageing human population is prone to both an increase in cardiovascular disease and to complications during and after infection with seasonal and avian influenza viruses [34,35], further exploration of the interplay between influenza and hemostasis would be of great interest. Most of the associations found in Table 2 show positive correlations between coagulation parameters and markers of inflammation (body weight decrease and relative lung weight increase). This comes as no surprise since the bidirectional cross-talk between coagulation and inflammation has been studied very well, whereby inflammation in general evokes a procoagulant response [36-38]. The specific disturbances in the tightly regulated balance between clotting, anti-coagulation and inflammation could be a target for novel intervention strategies in influenza. Following our observational study, an intervention model could further evaluate the role of coagulation in influenza virus pathogenesis and the potential processes for targeted intervention, for example by targeting protease receptor type-2 (PAR-2) activation in influenza pathogenesis. PAR-2 is an important receptor in both inflammation and coagulation, and recently described to have a major role in the damage seen after the inflammatory response during influenza virus infection [39,40]. While statins may also be interesting candidates for future studies. Statins may counteract specific inflammatory responses such as seen after acute coronary syndrome, and thereby may decrease mortality when given to influenza patients. Studying the influence of statin treatment on the procoagulant changes during influenza virus infection and the role these changes have in the postulated increased risk of myocardial infarction would be of great interest [41-43].

Collectively the data generated by our study will pave the way for further exploration of novel treatment and intervention strategies for influenza and its complications. Furthermore, based on the correlation between the viral infection - and coagulation parameters in this experiment, coagulation tests could serve as valuable biomarkers predicting disease severity. The ferret model likely offers the best opportunity to explore these options in a preclinical setting optionally also linked to host genetics since ferrets represent an outbred population.

## Conclusion

To our knowledge this is the first study that visualized hemostatic alterations in influenza virus infection in a controlled animal model resembling human disease. The drastic changes seen in a very short time period might be the result of consumptive coagulopathy. Interestingly even in the seasonal influenza group, with only relatively mild clinical ‘flu’ symptoms, infection had significant effects on systemic hemostasis. These results might help in further understanding the role of influenza infection in acute cardiovascular disease, while future research could indicate if alterations in coagulation have an important role in influenza pathogenesis.

## Methods

### Experimental design

Samples from 104, 11-month old, male, outbred ferrets (*Mustela putorius furo*) were used for this experiment as described previously [21]. Animals were inoculated both intratracheally and intranasally with one of three influenza viruses, or with control material (mock). All three influenza virus strains had been directly derived from patient isolates. For seasonal influenza, H3N2 virus (A/Netherlands/177/2008) [18], for pandemic influenza, pH1N1 influenza virus (A/Netherlands/602/2009) [44] and for highly pathogenic avian influenza virus (HPAI) the H5N1 strain (A/Indonesia/5/2005) were used [45]. Virus stocks were passaged three times in Madin-Darby Canine Kidney (MDCK) cells and titrated according to standard methods. The viruses were clarified and reached an infectious virus titer of 10^7.4^ median tissue culture infectious dose (TCID_50_) per ml for H3N2 virus, and 10^7.8^ TCID_50_ for both pH1N1 and HPAI-H5N1 virus [46]. The inoculum of the control group consisted of MDCK culture derived material which had been subjected to the same procedure to control for respiratory tract damage not related to replicating virus [21]. Inocula consisted of 3 mL volumes of virus preparations with 10^6^ TCID_50_ given per animal partly intratracheally and partly intranasally. Ferrets were randomly selected for any of the predefined time points before the start of the experiment. Four ferrets were euthanized per time point. Each ferret was sampled twice: before inoculation and when sacrificed. This resulted in 104 samples analyzed before inoculation (28 mock, 28 H3N2, 28 pH1N1 and 20 H5N1) and 4 samples per virus per time point (Table 4). During euthanasia, citrated blood was drawn by cardiac puncture in 3 mL citrate tubes and plasma was prepared for testing in coagulation assays.

**Table 4:** Distribution of the ferrets used in this study

**Group**	**P.I.**	**½ dpi**	**1 dpi**	**2 dpi**	**3 dpi**	**4 dpi**	**7 dpi**	**14 dpi**	**X**
Mock	28	4	4	4	4	4	4	4	
H3N2	28	4	4	4	4	4	4	4	
pH1N1	28	4	4	4	4	4	4	4	
H5N1	20	4	4	4	4	4	0	0	
Total	104	16	16	16	16	16	12	12	
Z									Y

Ferrets were sampled before inoculation with a mock control suspension, H3N2-, pH1N1- or H5N1 influenza virus. Coagulation analysis values from the different groups were compared using Mann–Whitney U test (X to Y). Subsequently values from the predefined timepoints were analyzed with the pre inoculation (P.I.) values using paired t-test (Y to Z).

### Ethics statement

To reduce the numbers of experimental animals used, we combined the earlier published influenza pathogenesis study [21] with the current study addressing questions related to activation of coagulation and tissue fibrin deposition during influenza virus infection. Animal housing and experiments were all in compliance with European guidelines (EU directive on animal testing 86/609/EEC) and Dutch legislation (Experiments on Animals Act, 1997) as documented previously [21]. The study protocol was approved by the independent animal experimentation ethical review committee of the Netherlands Vaccine Institute (permit number 200900201). Animal welfare was observed on a daily basis, and animal handling was performed under light anesthesia using a mixture of ketamine and medetomidine. After handling, atipamezole was administered to antagonize the effect of medetomidine.

### Coagulation assays

Prothrombin time (PT) and activated partial thromboplastin time (APTT) were measured using a BCS-XP coagulation analyzer (Siemens Healthcare Diagnostics) according to the instructions of the manufacturer. Clotting was initiated with Thromborel S (PT) and Pathrombin SL (APTT). VWF ristocetin cofactor activity was also determined on the BCS-XP with reagents of the manufacturer, and was expressed as percentage of normal pooled human plasma. Thrombin-antithrombin complexes (TAT, Siemens Healthcare Diagnostics) and D-dimer levels (Asserachrom, Roche, The Netherlands) were measured using enzyme-linked immunosorbent assay. All these assays were carried out within the BSL-3 setting after careful calibration and validation.

### Pathology and fibrin staining

Gross pathology and histopathology were evaluated as previously described [21]. Relative lung weight was used as a validated measure of gross pathology and lung inflammation [47]. For detection of fibrin, tissues were stained with the Lendrum staining according manufacturers’ protocol (MSB RRSK2-100 stain kit, Atom scientific). On each slide a small piece of human placenta was added as a positive control. Semi-quantitative assessment of fibrin expression in the lungs was performed as follows: for the alveoli, 25 arbitrarily chosen, 20x objective, fields of lung parenchyma of one lung section were examined by light microscopy for the presence of fibrin, without the knowledge of the identity of the animals. The scores (+ or -) were multiplied by 4 and presented as percentage.

### Virology

The presence of virus and virus replication in the respiratory tract were measured by determining infectious virus titers at different sites of the upper respiratory tract (URT) and lower respiratory tract (LRT). These results were combined with data retrieved by measuring viral antigen expression using standardized semi-quantitative immunohistochemistry carried out at different sites of the LRT as described previously [21].

### Statistics

All statistical analyses were performed using the software SPSS PASW statistics 17.0 and GraphPad Prism 4.01 for Windows. The data were expressed as mean or median with or without standard deviation or 95% confidence interval as described in figure and table legends. The compared groups are summarized in Table 4. The means per time point between the influenza virus infected groups and the mock control infected group were analyzed using the Mann–Whitney U test. Furthermore, values at the predefined time point of euthanasia were compared with pre-inoculation samples using paired t-testing. Differences with *p* ≤ 0.05 were considered statistically significant. For comparison of individual association between virological parameters and coagulation markers we used Pearson correlation coefficient, and transformed to match a normal distribution if needed. For correlation analysis we used Bonferroni correction for multivariable comparison setting p-value threshold to *p* ≤ 0.01.

## Competing interest

A. Osterhaus is a consultant to Viroclinics Biosciences BV, a spin out of Erasmus MC. The authors declare no conflicts of interest.

## Authors’ contributions

MG: Concept and design, executing experiments, analysis and interpetation of the data, writing of manuscript. ECMvG: Concept and design, interpretation of data, critical writing and revising of the manuscript and final approval of the manuscript. JMAvdB: Analysis and interpretation of data, critical writing and revising, final approval of manuscript. KS and KB: Executing experiments, analysis of data, approval of manuscript. JJTHR: Analysis and interpretation of data, approval of manuscript. GvA: Executing experiments, analysis and interpretation of data. TK: Interpretation of data approval of manuscript. BEEM: Interpretation of data, critical writing and revising of the manuscript and final approval of the manuscript. JCMM and ADMEO: Concept and design, analysis and interpretation of data, critical writing and revising of the manuscript and final approval of the manuscript.All authors read and approved the final manuscript.
